# Plant leaf tooth feature extraction

**DOI:** 10.1371/journal.pone.0204714

**Published:** 2019-02-13

**Authors:** Hu Wang, Di Tian, Chu Li, Yan Tian, Haoyu Zhou

**Affiliations:** 1 China Shipbuilding Industry Corporation, No. 722 Research Institute, Wuhan, Peoples Republic of China; 2 Faculty of Information Science and Technology, Wenhua College, Wuhan, Peoples Republic of China; 3 Electrical and Information Engineering Department, Huazhong University of Science and Technology, Wuhan, Peoples Republic of China; Georgia State University, UNITED STATES

## Abstract

Leaf tooth can indicate several systematically informative features and is extremely useful for circumscribing fossil leaf taxa. Moreover, it can help discriminate species or even higher taxa accurately. Previous studies extract features that are not strictly defined in botany; therefore, a uniform standard to compare the accuracies of various feature extraction methods cannot be used. For efficient and automatic retrieval of plant leaves from a leaf database, in this study, we propose an image-based description and measurement of leaf teeth by referring to the leaf structure classification system in botany. First, image preprocessing is carried out to obtain a binary map of plant leaves. Then, corner detection based on the curvature scale-space (CSS) algorithm is used to extract the inflection point from the edges; next, the leaf tooth apex is extracted by screening the convex points; then, according to the definition of the leaf structure, the characteristics of the leaf teeth are described and measured in terms of number of orders of teeth, tooth spacing, number of teeth, sinus shape, and tooth shape. In this manner, data extracted from the algorithm can not only be used to classify plants, but also provide scientific and standardized data to understand the history of plant evolution. Finally, to verify the effectiveness of the extraction method, we used simple linear discriminant analysis and multiclass support vector machine to classify leaves. The results show that the proposed method achieves high accuracy that is superior to that of other methods.

## Introduction

The potential value of leaf structure research in plant systematics, conservation biology, paleobotany, ecology, and paleoecology has begun to attract attention [1]. Generally, leaf structure allows closely related taxa to be distinguished from one another [2][3][4]. Moreover, it is found that the leaf architectural characteristics within the framework of molecular phylogenetic analysis can further highlight some evolutionary trends across angiosperms [5]. According to the biological characteristics of plant leaves, leaf structure has been examined and refined, and a relatively complete leaf structure system has been formed. In 2012, the Manual of Leaf Architecture provided a clearly defined and legendary terminology system for related research that could support wider use of leaf structure characters [6]. As one of the characteristics of plant leaves, leaf tooth is an important basis for the classification of plant leaf structure. A leaf tooth contains many characteristics of plant systematics and are extremely useful for circumscribing fossil leaf taxa [5][7][8]. Their prevalence in fossil floras provides reliable proxy data about pre-Quaternary terrestrial paleotemperatures [9][10][11]. Early studies have shown that species in colder regions have more leaf teeth, while plant species in warmer regions have smoother leaf edges and fewer leaf teeth [12–15]. For leaf specimens with intact leaf margins, the leaf features extracted in this study can be used as a “thermometer” to help simulate past climate [10][16].

Research on leaf features is basically divided into two categories: leaf shape and leaf margin (leaf edges). In a study on leaf shape, Xiaofeng et al. used Hu geometric moment and Zernike vertical distance to measure the shape of an input leaf image for automatic leaf classification [17]; Du et al. extracted eight characteristics such as the aspect ratio, rectangular and convex area of the leaf, convex perimeter ratio, sphericity, roundness, eccentricity, and shape factor [18]. In a study on leaf edge, Zheng et al. extracted the leaf edge by using the Harris and SUSAN algorithm and calculated three characteristic parameters: leaf edge sawtooth number, sharpness, and skewness [19]; Corney automatically acquired the shape and size of the leaf tooth by identifying the tooth on the leaf edge [20]; Jin proposed a method for classifying plant leaves using the sparse matrix of leaf tooth features [21]; Chen used the ratio between the internal distance of the leaf and the Euclidean distance to represent the local concavity and convexity of leaves [22], and classified the whole edge, tooth edge, wave edge, and leaf crack edge; Li used data dimensionality and character weighted semi-supervised clustering algorithm to identify the leaf by synthesizing leaf shape, leaf edge, texture, and other characteristics, which could be used for several applications [23]. In addition, several scholars have studied the extraction and classification of the non-botanical characteristics of leaves. Rahmani et al. studied the distance between the center of mass and the contour of the leaf margin and the texture features of the leaves. Then, they used a KNN classifier to classify the features of 1600 samples, with good accuracy [24]. Tsolaidis et al. extracted the features of the HOG and Zernike moments of 40 leaf samples from the Flavia and Swedish datasets, and classified the leaves with the extracted features using SVM. They achieved good results [25].

It should be noted that most of the extracted features are not strictly defined in botany, resulting in the inability to adopt a uniform standard to compare the accuracies of various feature extraction methods. In contrast to existing methods, in this study, the leaf tooth characteristics of plants are described and measured as per the botanical definition in the Manual of Leaf Architecture to provide reliable and effective features for automatic classification of plants. The basic process of the algorithm in this study is as follows: first, the leaf image is preprocessed to perform smoothing and denoising to obtain a better target image. Next, the curvature scale-space (CSS) [26] algorithm is used to extract the teeth. Based on this procedure, a method for detecting the edge of a leaf is proposed, and features such as number of orders of teeth, tooth spacing, number of teeth, sinus shape, and tooth shape are extracted according to their definition in the Manual of Leaf Architecture. The features are also extracted one by one. The data obtained through the algorithm can not only be used to classify plants, but also provide scientific and standardized data to determine the history of plant evolution.

## Proposed framework

**Fig 1:**
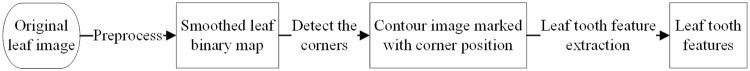
Block diagram of proposed framework for leaf tooth feature extraction. http://dx.doi.org/10.17504/protocols.io.wrxfd7n [PROTOCOL DOI].

The proposed algorithm framework presented in Fig 1 was used to extract the leaf tooth features based on the image in this study.

As shown in Fig 1, the overall algorithm framework is divided into three parts: preprocessing, corner detection, and leaf tooth feature extraction. Preprocessing is employed to obtain the binary image of the leaf, which includes image graying, image segmentation, and image smoothing. Corner detection is used to extract the key points (the leaf tooth convex or concave points) of the leaf edge for description and measurement of the leaf tooth features. Finally, leaf tooth feature extraction is defined according to the Manual of Leaf Architecture, and features such as number of orders of teeth, tooth spacing, number of teeth, sinus shape, and tooth shape of the leaf teeth are extracted. The preprocessing phase is divided into three stages. The techniques involved are described below.

The extraction of the leaf tooth does not depend on the color of the leaf; therefore, the acquired color image is first converted to a grayscale image. The color image has three channels, R, G, and B, while the grayscale image has only one channel. After the image is grayed out, the overall and partial chromaticity and brightness of the image can be better maintained, and the amount of calculation can be drastically reduced. In this study, we use the weighted average method to convert the image to grayscale [27]. The weighted sum of the three channels of color images is expressed as follows:

f(i,j)=0.299R(i,j)+0.587G(i,j)+0.114B(i,j) (1)

The purpose of image segmentation is to separate the leaves from the background to form a binary image for subsequent calculation of shape and texture characters. Here, the maximum interclass variance method (abbreviated as OTSU) is used to binarize the image [28]. The principle of this method is mainly to distinguish the background and target in the image by the grayscale characteristics of the image, and to calculate the interclass variance of the background and the target. The larger the variance, the greater the discrimination between the background and the target. Therefore, the segmentation with the largest variance between classes has the least probability of resulting in a misclassification.

After the image is binarized, there may be voids and breaks in the image, which may hinder the integrity of leaf extraction. Therefore, closing operations are performed to fill small holes and gaps in the image, and finally the image is smoothed and denoised. The closed operation used in our study is expressed as follows:

f·b=(f⊕b)⊖b (2)

Here, the radius of the disc structure element b is 2. This parameter was set after comparing the multi-group experimental effects. Using the parameters for closed operation can result in a good filling effect for tiny fractures (the leaf edge will have no obvious distortion), and also good robustness.

In corner detection based on the contour curve, methods based on calculation of the corner intensity are simple, but they are susceptible to noise interference. Methods based on calculation of the extreme curvature of the curve consider the local characteristics of corner points, but do not consider the global characteristics. This can result in the phenomenon of missed detection. To overcome these problems and achieve higher accuracy, a corner detection algorithm based on multiscale curvature space [26] is used to extract the leaf teeth, and a method for detecting the concave and convex points of a leaf edge is proposed based on this. Further, extraction of features including the number of orders of teeth, tooth spacing, number of teeth, sinus shape, and tooth shape of the leaf teeth is implemented according to their definition in the Manual of Leaf Architecture.

## Leaf tooth extraction algorithm

**Fig 2:**
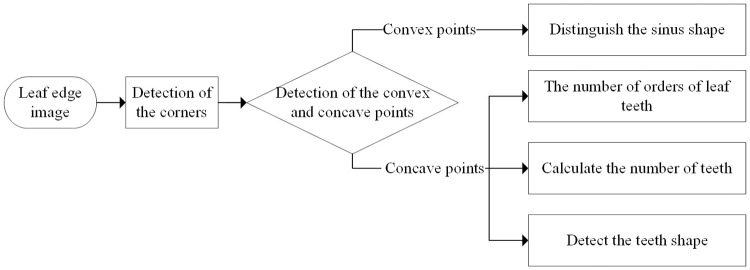
Flowchart of the leaf tooth feature extraction scheme.

As extracting the features of leaf teeth is the main focus of this study, a method of extracting leaf teeth was developed according to the characteristics of plant leaves. Unlike other parts of the leaf, the characteristics of the leaf teeth are reflected in the particularity of the morphological structure, that is, leaf teeth have obvious convex and concave points, which appear as corner points on the image. Therefore, a general grading and stepwise refinement method to extract the leaf teeth can be adopted. First, the corner points (both convex and concave points in the leaf teeth) are extracted from the leaf image, and then the two are further identified according to the different characteristics of convex and concave points. The basic flowchart is shown in Fig 2.

### Corner detection algorithm based on multiscale curvature space

**Fig 3:**
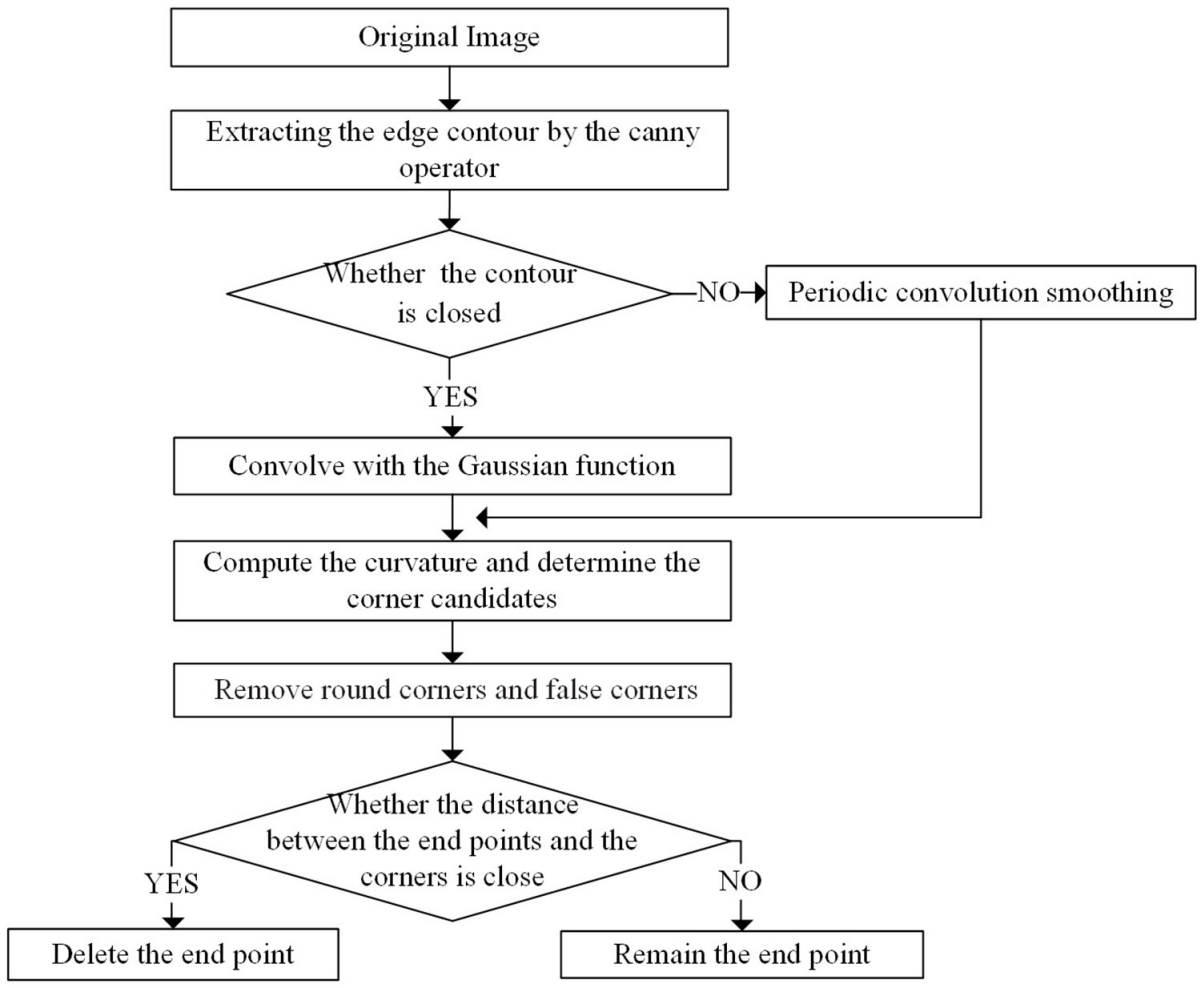
Flowchart of the CSS algorithm.

As mentioned above, as a result of the good performance of the multiscale space corner detection algorithm proposed in [26], it was used for leaf corner detection. The algorithm proceeds as follows: (1) Following preprocessing of the image, the Canny operator is used to extract the leaf edge. (The setting of the two thresholds (H and L) is explained in [29].) (2) After single pixelization of the leaf edges, the open-close situation of the contour curve is judged according to the preset threshold (Gap_size), then periodic convolution smoothing is used for the closed curve, and the open curve is convolved with the Gaussian function with width controlled by *δ*. (3) The curvature of all points on the curve is then calculated, and the point with the maximum local curvature value is defined as the corner candidates. (4) The threshold value C of the round corners removal is adaptively calculated according to the average curvature in the ROS region, and corners with curvature less than C are removed [30]. (5) The angle of the remaining corner candidates is calculated based on the recalculated dynamic support region, and corner candidates with angle smaller than the preset parameter T_angle are removed (removing the false corner). (6) The positions of the corner points are compared with the endpoints of the open curve and, if they are far apart, the endpoints are marked as candidate corners as well. The basic flowchart is shown in Fig 3.

**Fig 4:**
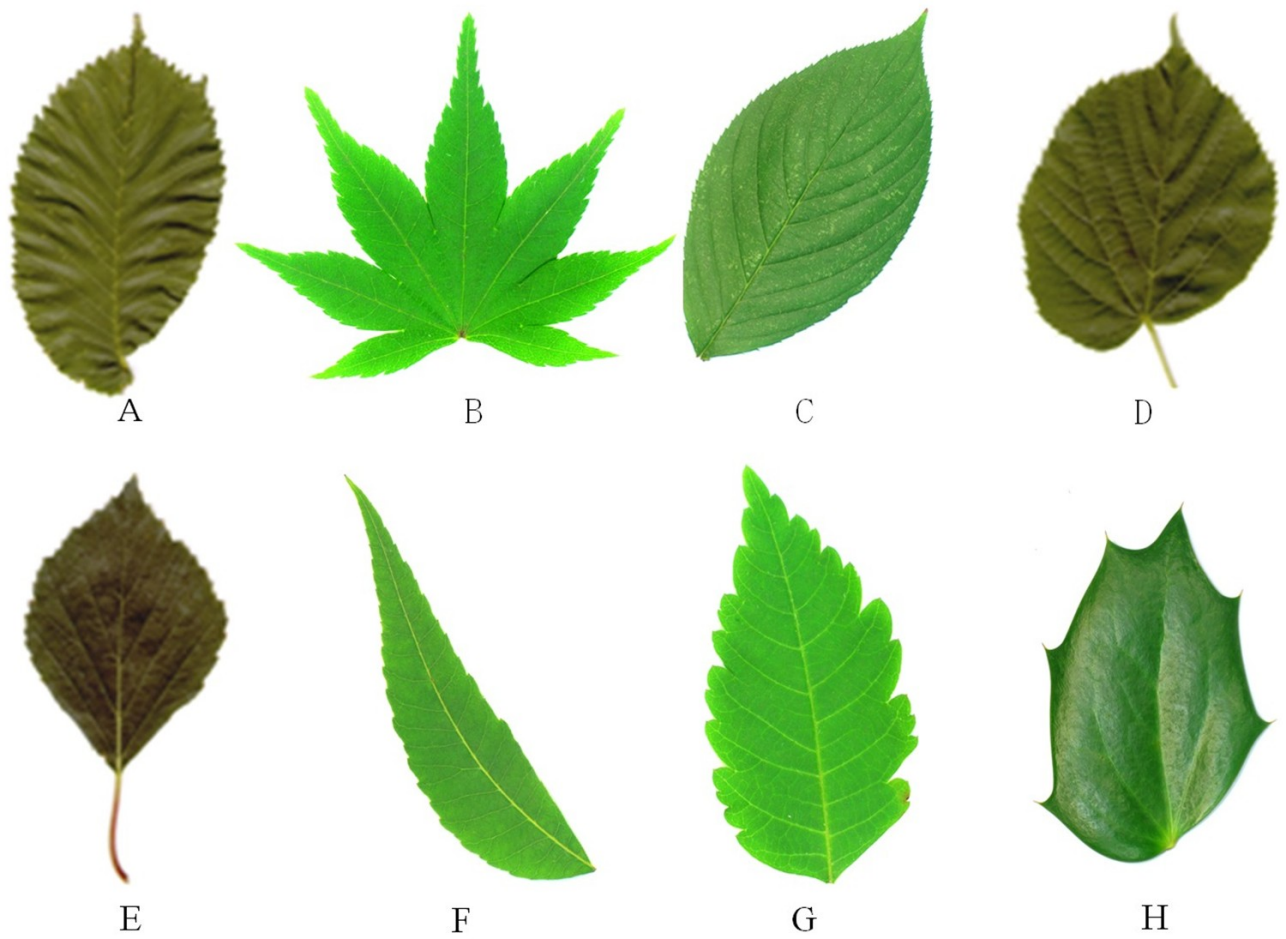
The eight types of leaves.

The parameters involved in the extraction of the leaf tooth feature are H, L, Gap_size, C, T_angle, and endpoint. Parameters such as H, L, Gap_size, T_angle, C, and Endpoint directly refer to the relevant parameters in [26] [29–31], where their robustness in the algorithm has been discussed in detail. The values are as follows: C = 1.5; T_angle = 162; sig = 4; H = 0.35; L = 0; Endpoint = 1; and Gap_size = 3. Because the value of threshold *δ* has the greatest influence on the extraction of the leaf teeth using this method, we tested the detection of the leaf teeth for eight types of leaves (as shown in Fig 4) with threshold values of 2, 4, 5, 6, and 7 (the leaf teeth of a and b are the most dense, whereas those of g and h are the most sparse). See Table 1 for details.

**Table 1:** Relationship between the number of extracted leaf teeth and the value of *δ*.

Leaf	A	B	C	D	E	F	G	H
*δ* **= 2**	210	134	93	54	29	34	30	10
*δ* **= 4**	164	115	90	52	31	30	25	7
*δ* **= 5**	135	99	78	43	24	28	25	7
*δ* **= 6**	102	78	67	39	21	25	23	6
*δ* **= 7**	85	68	57	18	19	20	23	6
**Manual observation**	136	104	82	53	36	30	23	6

It can be seen from Table 1 that the value of *δ* affects the accuracy of leaf tooth extraction. The number of leaf teeth detected by this method decreases as the value of *δ* increases. For different types of leaves, the value of *δ*is different corresponding to the smallest extraction error. For example, leaves a and b with dense teeth have the highest accuracy at *δ* = 5, and leaves g and h with sparse numbers of teeth have the highest accuracy at *δ* = 6. That is, for leaves with dense teeth, the leaf tooth point is captured more accurately when the curvature scale is small, and for leaves with sparse teeth, when the curvature scale is large, a part of the noise can be shielded to obtain a more precise leaf tooth point.

Studies have shown that the number of leaf teeth is related to the climate temperature, and the higher the temperature, the smaller the number of teeth [20]. In actual cases, it is generally easier to obtain information such as the temperature of the test sample growing place. Therefore, this priori information can be used to estimate the range of the number of leaf teeth and set the δ value. The number of teeth of most of the leaves used for testing was distributed between 25 and 90 in experiment 1. To minimize the extraction error of the number of teeth, a value δ = 4 was used in all the experiments presented in this study.

### Extraction of convex and concave points of leaf tooth

As for extraction of the concave and convex points of leaf teeth, the most direct method is to use the curvature feature to extract them directly. In this study, a robust extraction method for the concave-convex points of leaf teeth is proposed. The procedure is as follows: first, all the corner points are detected as the candidate points by the multiscale curvature spatial corner detection method introduced in Section 3.1. Second, considering the detected corner points may contain concave points and non-convex non-concave points, the convex points must be detected based on the different characteristics of these three types of points.

**Fig 5:**
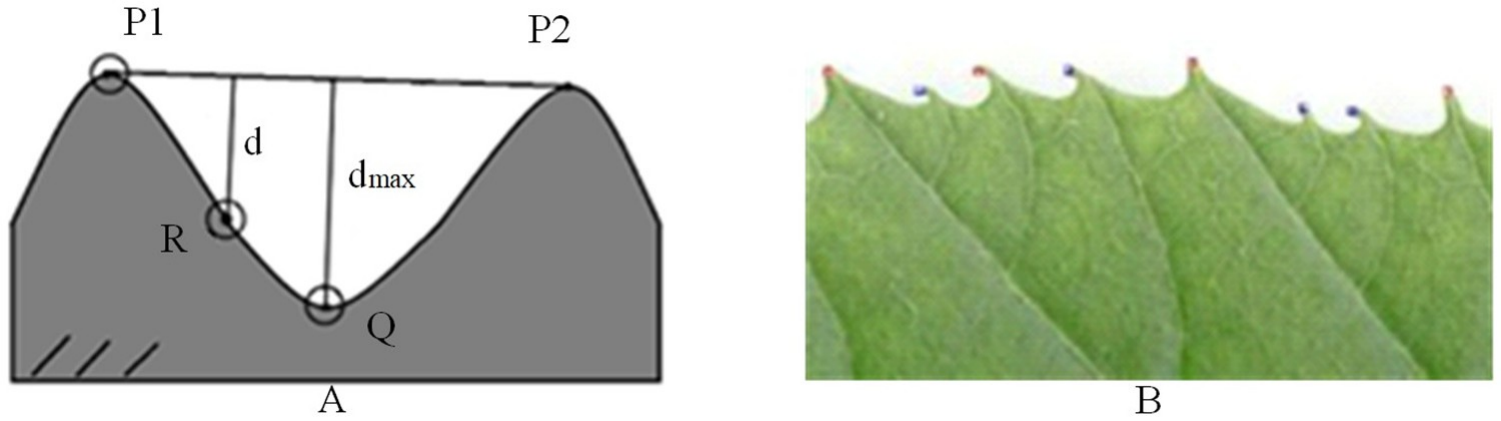
Example of convex points calibrated by the algorithm. (A) Schematic diagram of corner detection of leaf edge. (B) Example of the convex points of the algorithm calibration

The specific detection method of the convex points is as follows:

1. Use the CSS algorithm to detect all the corner points of the leaf;
2. With the corner point as the center, set the radius to L, and calculate the number of target points and background points falling in the circle. If the number of target points is less than that of the background points, it is a convex point. As shown in Fig 5A, P_1_ is a convex point, and Fig 5B is the actual image with convex points calibrated by the algorithm.

As shown in Fig 5A, the method of extracting the leaf tooth concave points is as follows: P_1_ and P_2_ are two adjacent convex points, the concave point Q will be between the two convex points, and the distance d_max_ between Q and the line P_1_P_2_ is inevitably greater than the distance d between the other points on the curve between P1 and P2. Therefore, by calculating the distance d from the point on the curve to the straight line determined by the two adjacent convex points, and comparing it, point Q corresponding to the maximum distance d_max_ is found to be a concave point.

## Extraction of leaf tooth features

According to the leaf structure classification system, the characteristics of the leaf teeth can be described by the number of orders of teeth, tooth spacing, number of teeth, sinus shape, and tooth shape. The description and extraction methods of these features will be given one by one.

### Leaf tooth feature description

**Fig 6:**
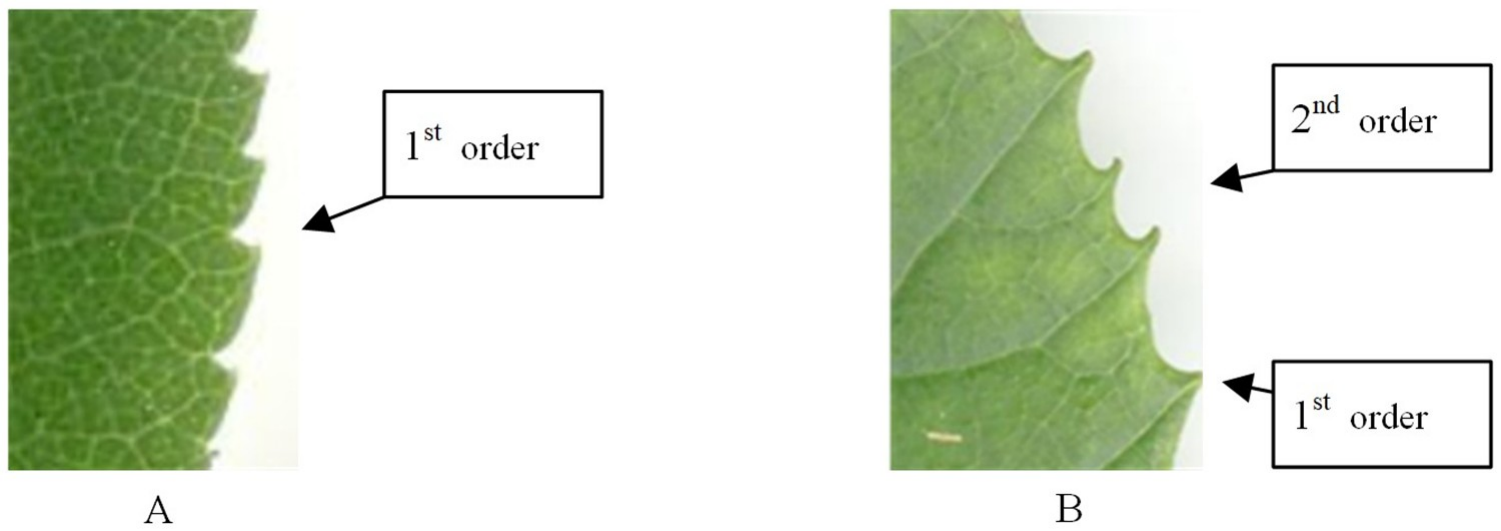
Schematic diagram of the orders of leaf tooth. (A) One order leaf tooth. (B) Two orders leaf tooth

**Fig 7:**
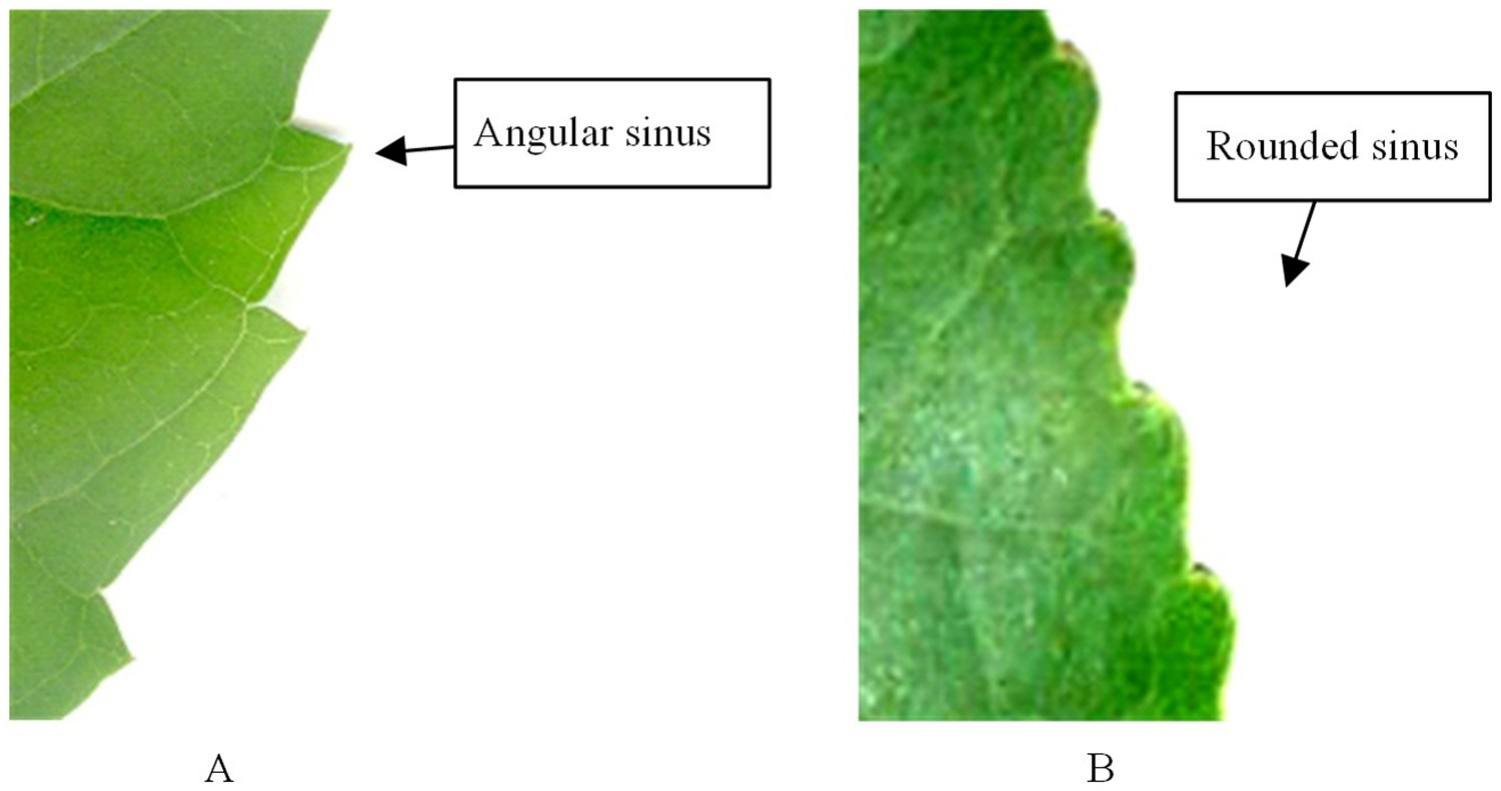
Sinus shape.

**Fig 8:**
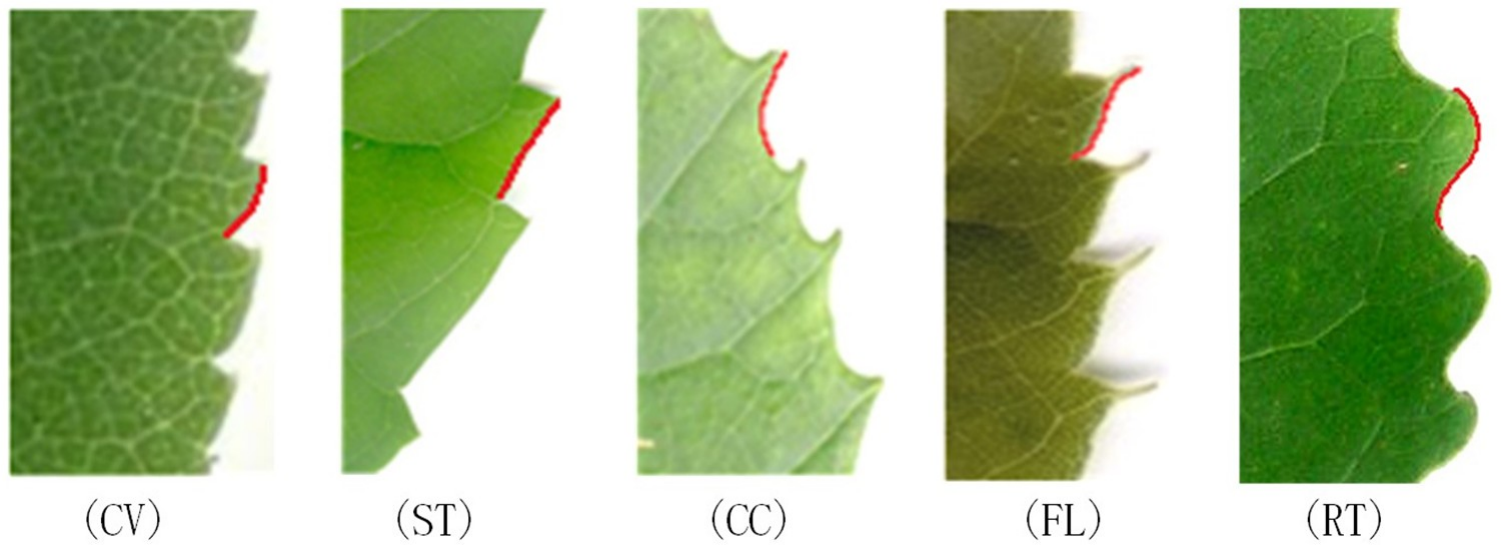
Chart of possible tooth shapes.

1. The number of orders of teeth is the number of discrete sizes of teeth. Order of one means that all teeth have the same or continuous change in size, as shown in Fig 6A. Order of two means teeth have two distinct sizes, as shown in Fig 6B. As the definition of 3rd order teeth in the Manual of Leaf Architecture is ambiguous, 3rd order teeth were not considered in the algorithm in this study.

1. The tooth spacing refers to the distance between adjacent teeth. Here, we simply perform qualitative analysis, not quantitative analysis; if the minimum intertooth distance > 60% of the maximum intertooth distance [32], the tooth spacing is regular, otherwise it is irregular [6].
2. The number of teeth is the total number of teeth on a leaf.
3. Sinus shape is the shape of a concave edge on the leaf, with angular or rounded shape, as shown in Fig 7.
4. The tooth shape is the distal and proximal flank curvatures relative to the midline of the tooth. The following states and abbreviations are used: convex (cv), straight (st), concave (cc), flexuous (fl), and retroflexed (rt) [6], as shown in Fig 8.

### Leaf tooth feature extraction

According to the concave and convex points detection method proposed in Section 3.2 and the definition of leaf tooth feature in Section 4.1, the implementation method for extracting leaf tooth feature, was as follows:

#### Number of orders of teeth

1. Calculate the distance from the convex point to the line formed by adjacent concave points to indicate the length of the teeth;
2. Normalize the teeth length, that is, divide the distance by the length of the longest tooth in the leaf (after removing abnormal points, which have values greater than twice the mean of the teeth length);
3. After normalization, the number of teeth for which the tooth length is less than the half of the maximum length are counted. If they are greater than half of the total number of leaf teeth, the leaf is considered to have two orders of teeth, otherwise it is considered to have the order of one;
4. If step 3 shows that the leaf has two orders of teeth, it is necessary to distinguish the first-order teeth and the second-order teeth by comparing the teeth length. The length of the first-order leaf teeth is significantly larger than the length of the two adjacent leaf teeth and the second-order teeth do not have this characteristic, the teeth can be distinguished.

#### Tooth spacing

1. Calculate the tooth spacing, that is, the distance between adjacent teeth;
2. Compare the minimum intertooth distance d_min_ with the maximum intertooth distance d_max_. If d_min_>(d_max_*0.6), the tooth spacing characters is regular; otherwise, it is irregular.

#### Tooth number

Calculate the total number of teeth of a leaf (i.e., the number of convex points using the abovementioned detection method).

#### Sinus shape

1. Calculate the curvature at the concave point of a leaf whose concave shape is rounded;
2. Calculate the curvature at the concave point of a leaf that has an angular concave shape;
3. According to the statistical data in steps 1 and 2, determine the curvature threshold value that distinguishes the angular Sinus and the rounded Sinus. This study calculated the sinuses curvature of many sample leaves (a total of 900 pieces in eight categories from the Swedish Leaf and Flavia datasets), and the mean curvature of all types of leaves, as shown in Table 2. The curvature of rounded sinuses was obtained as 0.009013–0.043190, while that of the angular sinuses was 0.061088–0.087059; hence, the threshold between the two sinus shapes can be set as 0.05, and can be used to determine the sinus shapes of other leaves without requiring alteration or re-training for different datasets.

**Table 2:** Mean curvature of leaves calculated in the sinus shape experiment.

Leaves	1	2	3	4	5	6	7	8
Means of curvature	0.087059	0.078781	0.065193	0.061088	0.043190	0.025086	0.016129	0.009013
Sinus shape	Angular	Angular	Angular	Angular	Rounded	Rounded	Rounded	Rounded

#### Tooth shape

**Fig 9:**
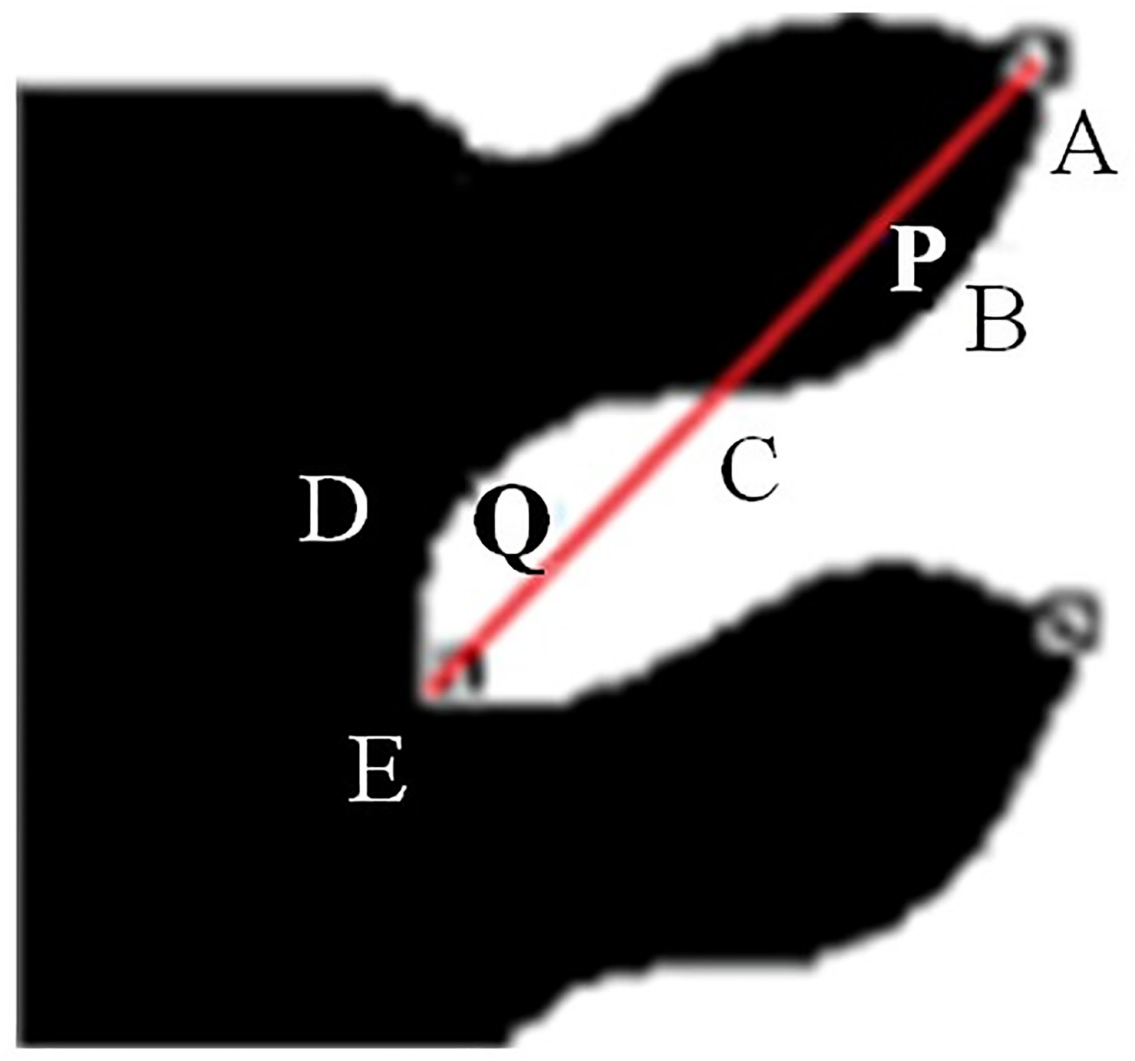
Tooth shape judgment diagram.

1. Extract the coordinates of an arc between a concave point and a convex point on the profile curve of the leaf tooth;
2. Fit the coordinates of the quadratic polynomial to obtain the corresponding polynomial;
3. Examine the quadratic parameter of the quadratic polynomial; if the parameter is equal to zero, it is straight; If the parameter is nonzero, the subsequent steps are taken to continue processing.
4. To further distinguish the tooth shape of CV, CC, FL, and RT, we use straight lines to connect the concavo-convex points, and count the number of black and white pixels in the closed area surrounded by the contour of leaf and straight line, and then judge the tooth shape according to the distribution of black and white pixels. As shown in Fig 9, A is the convex point of the leaf teeth, E is the concave point, and $\overset{⌢}{ABC\text{D}E}$ is the contour of the leaf, C is the intersection point of line AE and the contour (usually there is only one intersection point at most), B is any point on the curve $\overset{⌢}{AC}$, and D is any point on the curve $\overset{⌢}{CE}$.
5. If point C does not exist, count respectively the number of pixels with values of zero (black) and one (white) in the area of the line AE and the arc $\overset{⌢}{AE}$, and denote them as *n*_*B*_ and *n*_*W*_ respectively; if *n*_*B >*_ *n*_*D*_, the leaf tooth shape is CV; otherwise, it is CC.
6. If point C exists, count respectively the number of pixels with values of zero (black) and one (white) in the area (P) of the line AC and the arc $\overset{⌢}{ABC\text{D}E}$, and denote them respectively as *n*_*BB*_ and *n*_*BW*_; if *n*_*BB >*_ *n*_*BW*_, the area P is recorded as Black; otherwise, it is recorded as White. Then, count respectively the number of pixels with values of zero (black) and one (white) in the area (Q) of the line AC and the arc $\overset{⌢}{ABC\text{D}E}$, and denote them respectively as *n*_*DB*_ and *n*_*DW*_; if *n*_*DB >*_ *n*_*DW*_, the area Q is recorded as Black; otherwise, it is recorded as White. If P is Black and Q is White, the tooth shape is RT. If P is White and Q is Black, the tooth shape is FL.

## Experimental evaluation

### Experimental plan

To verify whether the proposed leaf structure feature description algorithm is scientific and effective, we implemented the algorithm using MATLAB 2017 (MathWorks, Natick, MA, USA) on a standard desktop PC (4.2 GHz CPU, 24 GB RAM). Processing of a single leaf took approximately 1.4 s. This could undoubtedly be improved through further optimization and/or using parallel computing. In this section, we provide relevant experimental results and analyze them.

**Fig 10:**
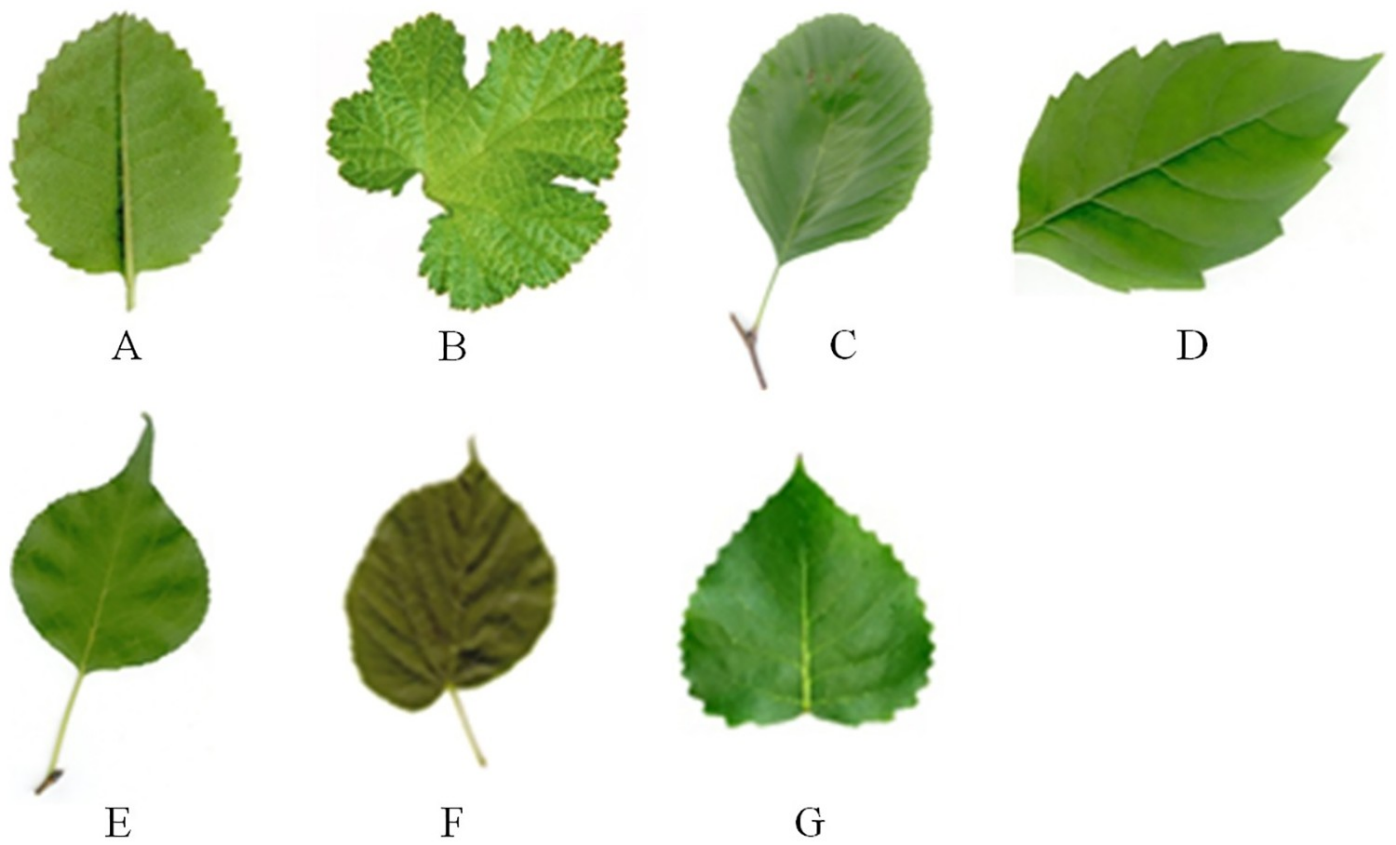
Examples of test leaves.

The test data were obtained from standard plant leaf databases (Swedish Leaf dataset, Flavia dataset, and Institute of Botany, Chinese Academy of Sciences) [33], and several representative shaped leaves were selected, as shown in Fig 10.

The extraction algorithm proposed in Sections 3 and 4 in this paper was used to extract the tooth features. We verify the validity of features extracted in two ways. The first method uses a manual view (Experiment 1) to verify whether the features extracted by the method are consistent with the actual characters of the leaf; the second method applies the extracted features to a classification algorithm (Experiment 2) to indirectly verify the validity of the features by classification; the classification is performed using a general evaluation method: confusion matrix and Kappa statistics. The confusion matrix consists of four values: true positive, false positive, false negative, and true negative. Based on these data, the value of true negative rate (specificity), negative predictive value (NPV), precision, recall, accuracy, F-measure, and Kappa coefficient (Kappa) are calculated [34]. Table 3 lists the Kappa coefficients for the classification, and the accuracy criteria are determined using the method proposed by Landis and Koch [35].

**Table 3:** Level of classification accuracy according to the Kappa coefficient value.

Kappa Coefficient (K)	Quality
K < 0.2	Poor
0.2 ≥ K < 0.4	Reasonable
0.4 ≥ K < 0.6	Good
0.6 ≥ K < 0.8	Very Good
K ≥ 0.8	Excellent

## Results and analysis

### Experiment 1

The experimental results of the first method (manual view) of the algorithm validation are shown in Table 4 through Table 6.

**Table 4:** Results of tooth extraction.

Category	Orders of teeth	Tooth space	Number of teeth	Sinus shape	tooth shape
**Leaf 1**	1	Regular	32	Angular sinus	CV.
**Leaf 2**	2	Irregular	82	Angular sinus	CV..
**Leaf 3**	2	Irregular	79	Rounded sinus	CC.
**Leaf 4**	1	Irregular	12	Angular sinus	ST.
**Leaf 5**	1	Irregular	84	Angular sinus	CV.
**Leaf 6**	2	Irregular	57	Angular sinus	FL.
**Leaf 7**	2	Irregular	23	Rounded sinus	RT.

**Table 5:** Accuracy statistics of the orders of leaf tooth.

Category	First-order teeth		Accuracy	Second-order teeth		Accuracy
	Positive	Negative		Positive	Negative	
**Leaf 1**	23	6	79%	-	-	-
**Leaf 2**	17	7	70%	42	16	72%
**Leaf 3**	33	1	97%	47	6	88%
**Leaf 4**	11	2	84%	-	-	-
**Leaf 5**	27	8	77%			
**Leaf 6**	15	2	88%	34	6	85%
**Leaf 7**	5	2	71%	15	6	60%

**Table 6:** Statistics of the curvature of the concave shape.

Groups	C1	C2	C3	C4	C5	C6	C7	C8	C N	Mean Value
**Rounded**	0.0665	0.1238	0.0641	0.0618	0.0509	0.1072	0.0620	0.1072	…	0.0673
**Angular**	0.1344	0.1806	0.2540	0.2649	0.1853	0.1764	0.1774	0.1905	…	0.2044

From the experimental results listed in Tables 3–5, it can be seen that the method accurately extracts the tooth characteristics of the leaf. Moreover, the tooth spacing, the number of teeth, the shape of the concave points, and the number of orders of teeth are also in line with the observed results, which indicates that the CSS method is accurate and reliable for the extraction of leaf teeth features. Simultaneous curvature is an effective method to describe the shape of leaf concave shape.

## Experiment 2

To further test the effectiveness of the extraction method proposed in this paper, the extracted features were used for leaf classification. Thus, the effectiveness of the method was verified in terms of classification accuracy. Because we use the same method to extract multiple features from the same sample, if the accuracy of the classification is higher, the features have a better distinction. An extraction method of leaf tooth features has been discussed in detail and the classification of leaves based on leaf teeth characteristics has been studied, with good results being achieved [20]. Other methods [21–23] use a combination of leaf tooth features and non-leaf tooth features. As the focus of this study was only on leaf teeth features, the comparison method was based on the selection proposed in [20]. A leaf identification method identifies features such as total area of teeth, internal angles, number of teeth, and total length of outer edges.

The original input uses four types of plant leaves, a total of 700 leaf images (from the Swedish Leaf Dataset [30]; the dataset contains scanned images of 15 leaves and the selected species have greater similarity, which is considered as challenging to be classified). We use our method to extract the leaf tooth features from the original leaves as an input dataset; we also need to divide the data into a non-overlapping training set (90% of the records) used to estimate the model parameters, and a testing set (10% of the records) used to estimate its accuracy.

First, we used multiclass linear discriminant analysis (LDA) to reduce the dimensions. Then, we used the shortest distance for classification and predicted the category labels. The feature classification test results extracted by the proposed method and the method proposed in [20] are shown in Table 7.

**Table 7:** LDA results.

Category	Method	Specificity	NPV	Precision	Recall	F1-measure	Accuracy	Kappa
**1**	Our method	0.2667	0.4444	0.3939	0.8667	0.5417	0.4167	0.2222
	Proposed in [20]	0.1333	0.4	0.2667	0.8	0.4	0.3	0.0667
**2**	Our method	0.5556	0.4464	0	0	-	0.4167	0.2222
	Proposed in [20]	0.3333	0.2885	0.375	0.2	0.2609	0.3	0.0667
**3**	Our method	0.3778	0.34	0.8	0.5333	0.64	0.4167	0.2222
	Proposed in [20]	0.3778	0.2931	0.5	0.0667	0.1176	0.3	0.0667
**4**	Our method	0.4667	0.4468	0.3077	0.2667	0.2857	0.4167	0.2222
	Proposed in [20]	0.3556	0.2909	0.4	0.1333	0.2	0.3	0.0667

It can be seen from Table 7 that the accuracy of leaf classification using the proposed method is 41.67%, and Kappa coefficient classified the result as “reasonable.” However, the accuracy of leaf classification is only 30% using the extraction method in [20] and the Kappa coefficient classified the result as “poor” according to the accuracy levels (K<0.2). Therefore, the feature extracted by the proposed method has higher accuracy for leaf classification; that is, the features extracted using this method have better discrimination capability for different leaves.

Second, we used a multiclass support vector machine (SVM) classifier to predict the category of leaves. The results of the feature classification test for the proposed method and the method proposed in [20] are shown in Table 8.

**Table 8:** SVM classification results.

Category	Method	Specificity	NPV	Precision	Recall	F1-measure	Accuracy	Kappa
**1**	Our method	0.8889	0.8511	1	0.8667	0.9286	0.8833	0.8444
	Proposed in [20]	0.2667	0.2791	0.1765	0.2	0.1875	0.25	0
**2**	Our method	0.8889	0.9302	0.7647	0.8667	0.8125	0.8833	0.8444
	Proposed in [20]	0.2444	0.2683	0.2105	0.2667	0.2353	0.25	0
**3**	Our method	0.8444	0.8444	1	1	1	0.8833	0.8444
	Proposed in [20]	0.1556	0.1944	0.3333	0.5333	0.4103	0.25	0
**4**	Our method	0.9111	0.9111	0.8	0.8	0.8	0.8833	0.8444
	Proposed in [20]	0.3333	0.25	-	0	-	0.25	0

It can be seen from Table 8 that the accuracy of leaf classification with the proposed extraction method is 88.33%, and the Kappa coefficient classified the solution as “excellent”; the accuracy of leaf classification is only 25% if we use the extraction method proposed in [20], and the 4th leaf is classified incorrectly, for which the Kappa coefficient classified the results as “poor.” This proves that the leaf tooth features as referred to leaf structure classification system in botany, such as number of orders of teeth, tooth spacing, tooth number, concave shape and so on, can accurately describe the differences between different types of plant leaves; the accuracy of SVM classification using this method is significantly higher than that using LDA method.

## Conclusion

The extraction of leaf features has important theoretical and practical significance in biological ecosystems and leaf retrieval. With the development of computer and image processing technology, automatic high-precision extraction of leaf structure has become an emerging interdisciplinary subject. For extracting leaf teeth characteristics, this study followed the biological leaf structure classification system; we focused on describing and extracting leaf teeth by identifying factors such as number of orders of leaf teeth, tooth spacing, number of teeth, sinus shape, and tooth shape. The experimental results showed that the leaf tooth description and measurement method proposed in this study can provide an effective way for a refined description of leaf features and leaf classification; however, the method is only applicable for plants with leaf teeth. For plants without leaf teeth, other features of leaves will need to be considered, which will also be the direction of our work in the next stage. For example, we will explore a method of automatic extraction of leaf-related features through image processing according to the description and definition of leaf organization, glands, petiole, and other features in the Manual of Leaf Architecture. These scientific and canonical leaf characterization data can be used to correlate and contrast plants at different times, thereby inferring the evolutionary history of plants.
